# Interaction of KLAKLAK-NH_2_ and Analogs with Biomimetic Membrane Models

**DOI:** 10.3390/pharmaceutics16030340

**Published:** 2024-02-28

**Authors:** Victoria Vitkova, Krassimira Antonova, Ognyan Petkov, Angelina Stoyanova-Ivanova, Sirine Jaber, Vladislava Ivanova, Emilia Naydenova, Dancho Danalev

**Affiliations:** 1 Georgi Nadjakov Institute of Solid State Physics, Bulgarian Academy of Sciences, 1784 Sofia, Bulgaria; krasa@issp.bas.bg (K.A.); okrpetkov@issp.bas.bg (O.P.); angelina@issp.bas.bg (A.S.-I.); 2 University of Chemical Technology and Metallurgy, 8 Kliment Ohridski Blvd., 1756 Sofia, Bulgaria; sirine@uctm.edu (S.J.); ivanova_vl@uctm.edu (V.I.); emilia@uctm.edu (E.N.)

**Keywords:** amphipathic peptides, lipid vesicles, bending elasticity, electrical capacitance, FTIR-ATR spectroscopy

## Abstract

Background: Specifically designed peptide mimetics offer higher selectivity regarding their toxicity to mammalian cells. In addition to the α-helix conformation, the specific activity is related to the peptide’s ability to penetrate the cell membrane. The alterations in lipid membrane properties were addressed in the presence of the peptide KLAKLAK-NH_2_ and analogs containing β-alanine, strengthening the antibacterial activity and/or naphtalimide with proven anticancer properties. Methods: The molecular interactions of the peptide mimetics with POPC bilayers were studied using FTIR-ATR spectroscopy. The thermal shape fluctuation analysis of quasispherical unilamellar vesicles was applied to probe the membrane bending elasticity. The impedance characteristics of bilayer lipid membranes were measured using fast Fourier-transform electrochemical impedance spectroscopy. Results: A lateral peptide association with the membrane is reported for β-alanine-containing peptides. The most pronounced membrane softening is found for the NphtG-KLβAKLβAK-NH_2_ analog containing both active groups that corroborate with the indications for 1,8-naphthalimide penetration in the lipid hydrophobic area obtained from the FTIR-ATR spectra analysis. The β-alanine substitution induces strong membrane-rigidifying properties even at very low concentrations of both β-alanine-containing peptides. Conclusions: The reported results are expected to advance the progress in tailoring the pharmacokinetic properties of antimicrobial peptides with strengthened stability towards enzymatic degradation. The investigation of the nonspecific interactions of peptides with model lipid membranes is featured as a useful tool to assess the antitumor and antimicrobial potential of new peptide mimetics.

## 1. Introduction

Nowadays, problems with bacterial resistance are reaching pandemic proportions. Many multidrug-resistant bacterial strains such as *Escherichia coli* (*E. coli*), *Pseudomonas*, and *Staphylococcus*, etc. have already proved their ability to find new pathways to overcome antibiotic action, and they are responsible for difficult-to-control bacterial infections. Thus, the problem with bacterial resistance against conventional medicaments becomes a serious challenge for medicine [1,2]. Currently, searching for new alternatives to using antibiotics to fight bacterial resistance is the main task of many scientific groups. Medical practice gradually began to rediscover the unused and not well-studied potential of natural biomolecules like amino acids, peptides, carbohydrates, etc. contained in different natural kingdoms. Specific and very useful features of peptide molecules are their natural mechanisms of elimination, small sizes and low or no side effects, etc. [3]. But, one of the most valuable properties is their ability to penetrate cell membranes. Thus, currently, they attract the attention of many scientific groups as a possible alternative to overcome bacterial resistance.

Many years ago, the discovery of magainins, a group of antimicrobial peptides (AMPs), initiated a deep search in the world of these natural molecules [4]. It was found that AMPs have a key modulatory role in the initial immune response of many organisms including plants, animals, insects, and a lot of bacteria [5,6,7,8], and they exhibit direct antimicrobial activity [9]. AMPs are characterized by a short amino acid chain including around 10 to 50 residues [10,11]. They are cationic, characterized generally by a lack of a stable conformation in water as well as by the ability to form amphipathic α-helixes in membranes. This specific feature helps them to disrupt the cell membranes of a large spectrum of bacteria, protozoa, and fungi [12]. Several mechanisms of penetration in the cell membrane are currently reported in the literature, such as the barrel-stave [13], toroid-pore, and the carpet-like model [14], etc. [15,16].

Javadpour et al. [17] reported the discovery of a new potential AMP named (KLAKLAK)_2_-NH_2_. Their investigations revealed that the peptide has both antimicrobial and anticancer properties due to a similar mechanism of penetration in both normal and tumor cells [17]. A conclusion made by Javadpour’s group is that the minimum-double repeating of the sequenced KLAKLAK is necessary for antibacterial activity. However, our previous studies on (KLAKLAK)_2_-NH_2_ analogs revealed that a single KLAKLAK-NH_2_ sequence has moderate activity against the Gram-negative test microorganism *E. coli* K12 407 at a 20 µM concentration, but its analogs containing unnatural amino acid β-Ala and nor-Leu in the primary structure do not show any activity [18]. In addition, if unnatural amino acids β-Ala and nor-Leu are added to the primary structure of double-sequenced analogues (KLβAKLβAK)_2_-NH_2_ and (Knor-LAKnor-LAK)_2_-NH_2_, antibacterial activity is enlarged against the Gram-positive microorganisms of the strain *Bacillus subtilis* 3562 [19,20]. Often, peptide molecules are used as vehicles in order to transport active molecules in the cell due to their ability to penetrate the membrane [21,22]. In addition, our studies showed that KLAKLAK-NH_2_ analogs could be successfully used as such as vehicles to transport a second pharmacophore, a very well-known naphtalimide with proven anticancer properties [23]. Moreover, both parts of the molecule showed a synergic antiproliferative effect [18,19].

Here, we study the ability of both shortened analogs KLAKLAK-NH_2_ and KLβAKLβAK-NH_2_ as well as those modified with the unnatural amino acid β-Ala doubled-sequenced analog (KLβAKLβAK)_2_-NH_2_ to influence the important mechanical and impedance parameters of model cell membranes and to predict their ability to penetrate the cell. We explore the alteration of membrane properties by the 1,8-naphtalimide- KLβAKLβAK-NH_2_ mimetic in order to unravel the membrane-related anticancer effects of this shortened analog. Apart from assuring cell integrity and compartmentalization, biomembranes are also involved in cellular processes and are related to strong membrane deformations and morphological changes. The investigation of the structural, mechanical and impedance properties of lipid bilayers builds the necessary background for revealing important functional parameters of membranes. We probed the mechanical and electrical characteristics of phosphatidylcholine membrane models to shed light on the effect of the studied peptide molecules. The bending elasticity was measured using the flicker spectroscopy of quasispherical giant unilamellar vesicles (GUVs) modelling the basic physical properties of biomembranes [24,25,26]. With characteristic diameters comparable to the typical cell sizes (5–100 µm), GUVs serve as a versatile platform for the direct visualization of numerous membrane-related phenomena [27]. FTIR-ATR measurements of liposome suspensions were carried out to unravel the molecular interactions of the studied peptide sequences with POPC bilayers [28,29,30,31].

## 2. Materials and Methods

### 2.1. Materials

**Figure 1:**
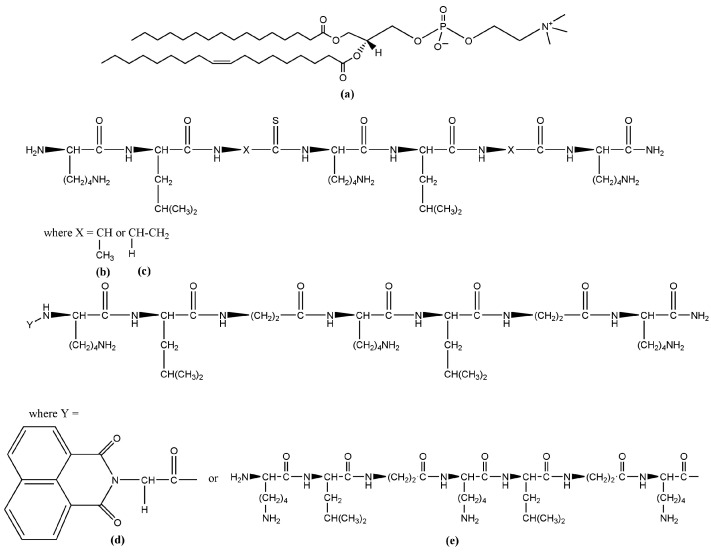
Chemical structures of the lipids and peptides used in the study: (**a**) 1-palmitoyl-2-oleoyl-glycero-3-phosphocholine (Avanti Polar Lipids, Alabaster, AL, USA); (**b**) KLAKLAK-NH_2_; (**c**) KLβAKLβAK-NH_2_; (**d**) NphtGKLβAKLβAK-NH_2_; and (**e**) (KLβAKLβAK)_2_-NH_2_.

The analytical-grade chemicals, which were necessary to prepare the membrane lipid systems studied here, were used as purchased. Vesicles and suspended lipid bilayers were produced from the synthetic phosphatidylcholine (Figure 1a) 1-palmitoyl-2-oleoyl-sn-glycero-3-phosphocholine (POPC, C_42_H_82_NO_8_P, 760.08 g/mol), provided by Avanti Polar Lipids (Alabaster, AL, USA). Organic solvents methanol and chloroform purchased from Sigma-Aldrich (Darmstadt, Germany) were used for the preparation of lipid solutions. For all suspensions, bi-distilled water from a quartz distiller, filtered through 0.2 µm sterile syringe filters (Corning Incorporated, Wiesbaden, Germany), was used. The four peptide sequences (Table 1 and Figure 1b–e) studied here, namely KLAKLAK-NH_2_ (C_36_H_71_N_11_O_7_) and its analogues NphtG-KLβAKLβAK-NH_2_ (C_50_H_78_N_12_O_10_), KLβAKLβAKNH_2_ (C_36_H_71_N_11_O_7_), and (KLβAKLβAK)_2_-NH_2_ (C_71_H_135_N_21_O_15_), were obtained by solid phase synthesis (SPPS) [9].

### 2.2. Methods

#### 2.2.1. Peptide Synthesis

KLAKLAK-NH_2_ and its analogues were obtained by solid phase synthesis (SPPS) as described in detail elsewhere [18]. Briefly, the Fmoc/O*t*-Bu strategy was used to bond subsequently all amino acids from the primary structure of the peptide, starting from C- to N-terminus by means of HBTU/DIPEA as condensation agents. PyBOP/DIPEA was used to introduce NphtG in the N-terminus of peptide P2. ^α^N-Fmoc-group was removed at every step by treatment with 20% piperidine in DMF. The reactions of coupling and deprotection were monitored by a standard Kaiser test [32]. The final product was removed from the resin by treatment with a mixture of 95% trifluoroacetic acid (TFA), 2.5% triisopropylsilane (TIS) and 2.5% water for 4 h. At the end of the reaction time, liquids were removed under vacuum, and crude oil was precipitated in cold ether. The purity of obtained peptides and their structure were analyzed using HPLC-MS. The chromatographic purity was 100% for peptides P1, P2 and P3-long and 99.14% for the peptide P3 [18,19].

#### 2.2.2. Vesicle Preparation

Giant unilamellar vesicles (GUVs) were produced from POPC and POPC-peptide mixtures using the electroformation method [33]. Two indium tin oxide (ITO)-coated glass plates with a polydimethylsiloxane (PDMS, Dow Corning, Wiesbaden, Germany) spacer with 5 mm-thickness comprised the electroformation cell. After spreading out 50 μg of POPC or POPC–peptide solution with total concentration of 1 g/L in chloroform-methanol solvent (9:1 volume parts) on the ITO-coated surface of each electrode, both were placed in a desiccator for a couple of hours under vacuum until the organic solvents completely evaporated. The electrodes were connected to a functional generator and the electroformation protocol [34] was applied immediately after filling the electroformation chamber with 0.2 µm-filtered bi-distilled water. The application of AC electric field (10 Hz; 0.4 V/mm peak-to-peak) was applied to the electroformation cell for a couple of hours to produce a high yield of quasispherical single-wall giant vesicles without membrane defects and appropriate for thermal shape fluctuation analysis.

Multilamellar vesicles (MLVs) were produced from POPC or POPC–peptide mixtures in bi-distilled water following the mechanical dispersion method [35,36,37]. Lipid or lipid–peptide mixtures dissolved in chloroform/methanol (9:1 volume parts) at 1 mg/mL were evaporated under vacuum for 5 h in order to obtain a solvent-free dry lipid film formed on the bottom of a glass vial. The lipid or lipid-peptide film so-obtained was hydrated using bi-distilled water to achieve 5 mg/mL as a final concentration of lipid or lipid–peptide mixture in the liposome suspension. After vortexing for 2 min, we placed each sample in an ultrasonic bath and left them to hydrate overnight. All peptide-containing samples were prepared with peptide-to-lipid molar ratio $C_{P}^{tot}/C_{L}^{tot}$ = 0.185. 

#### 2.2.3. Formation of Bilayer Lipid Membranes (BLMs)

We studied the influence of KLAKLAK-peptides on the impedance parameters of bilayer lipid membranes (BLMs) from POPC obtained by the Montal–Mueller approach [38,39]. The lipid was dissolved in HPLC-grade pentane CH_3_(CH_2_)_3_CH_3_ and hexane CH_3_(CH_2_)_4_CH_3_ (99:1, volume). Eastern Scientific LLC (Rockville, MD, USA) supplied us with the Montal–Mueller chamber (model BC-20A), in which we produced solvent-free bilayers suspended on the circular 100 µm aperture in a Teflon membrane with thickness of 0.025 mm following the established protocol [34,38]. The final bilayer formation was controlled electrically [38].

#### 2.2.4. Thermal Shape Fluctuation Analysis (TSFA) of GUVs

Two mechanical constants $k_{s}$ and $k_{c}$, corresponding to the resistance to stretching and bending, respectively, describe mechanically a lipid bilayer in its liquid state above the main transition temperature. The relative bilayer’s area changes $\Delta A/A_{0}$ and the membrane stretching modulus $k_{s}$ determine the mechanical tension $\sigma =k_{s}\Delta A/A_{0}$. In the present study, we measure the mechanical properties of symmetrical membranes bathed by identical aqueous solutions on both sides of the bilayer. Therefore, the bending modulus $k_{c}$ relates to the density of the bending energy $F_{c}$ by the equation $F_{c}=k_{c}{\left(c_{1}+c_{2}\right)}^{2}/\;2+k_{G}c_{1}c_{2}$, where $k_{G}$ stands for the saddle-splay (Gaussian) modulus and $\left(c_{1}+c_{2}\right)$ and $c_{1}c_{2}$ denote the total curvature and the Gaussian curvature, respectively [40]. 

We measured $k_{c}$ by thermal shape fluctuation analysis (TSFA) of quasispherical vesicles [41], based on Legendre analysis of the time-averaged angular autocorrelation function of the vesicle radius, ξ(γ) [42] with an amplitude $B_{n}\left(k_{c},\;\bar{\sigma }\right)$ of the n-th mode in its decomposition with respect to Legendre polynomials expressed with the bending constant $k_{c}$ and the reduced membrane tension $\bar{\sigma }=\sigma R_{0}^{2}/k_{c}$, where $R_{0}$ denotes the vesicle radius. A stroboscopic illumination of the observed vesicles, synchronized with the camera, is implemented in our TSFA set-up, which makes possible the recording and analysis of the fast fluctuation modes [41]. All measurements of the membrane-bending elasticity were conducted at (37 ± 0.1) °C with temperature control via a two-channel system operating both with the oil-immersed objective (100×, numerical aperture 1.25; 0.106 μm/pixel) and the plate of an inverted microscope Zeiss Axiovert 100 (Zeiss, Jena, Germany) equipped with a CCD camera (C3582, Hamamatsu Photonics, Shizuoka, Japan) connected to a frame grabber (DT3155, Data Translation, Marlborough, MA, USA, 768 × 576 8-bit pixels, 0.172 μm/pixel) for digitization and analysis. Only flaccid nearly spherical vesicles with diameters larger than 10 μm were recorded and subjected to TSFA. Several hundred images were captured once per second for the calculation of the membrane-bending constant and tension of every studied vesicle using an in-house non-commercial software as described in [41]. The contour corresponding to the equatorial cross-section of the membrane of a quasispherical vesicle observed by phase-contrast microscopy was digitized using a procedure reported in detail in [42,43]. The values of $k_{c}$ and $\bar{\sigma }$ for each studied vesicle were obtained by a fitting procedure performed as in [41,42,43]. Resulting from the stochastic background of vesicle formation, the membrane tension $\bar{\sigma }$ is different for each vesicle, whilst the value of $k_{c}$ quantifying the bending rigidity of membranes with the same composition and structure characterizes the entire vesicle collection in the sample. The statistical relevance of this assumption was determined by $\chi^{2}$-goodness of fit test. The below-reported values of the membrane-bending modulus were obtained over an ensemble of not less than six GUVs collected from at least two different preparations. 

#### 2.2.5. Fast Fourier-Transform Electrochemical Impedance Spectroscopy (FFT-EIS) of Lipid Bilayers

The effect of KLAKLAK-peptides on the impedance characteristics of solvent-free POPC BLMs was investigated by fast Fourier transform electrochemical impedance spectroscopy (FFT-EIS) immediately after BLM formation using equivalent circuit modelling as described in [34]. The measurement consisted of the application of a small-amplitude (~10 mV) multisine perturbation signal in the frequency range of 1.5 Hz–50 kHz. The acquisition procedure comprised the simultaneous recording of the perturbation and the response signals followed by fast Fourier transformation (FFT) to the frequency domain. Hence, the impedance spectrum was acquired in seconds by simultaneously monitoring the stationarity [43,44]. The latter excluded possible artifacts influencing the calculated impedance values. The capacitance $C_{BLM}$ and resistance $R_{BLM}$ of planar lipid bilayers were obtained from the analysis of the FFT-EIS data recorded and the equivalent circuit modeling the Montal–Mueller BLM configuration [34]. The specific capacitance $C_{m}=C_{BLM}/S_{m}$ and resistance $R_{m}=R_{BLM}S_{m}$ of bilayers were deduced from the values measured for the capacitance and resistance of the bilayer and the surface area $S_{m}=$ 8 × 10^−5^ cm^2^ of the 100 µm circle aperture on which BLMs were suspended. Further, we represent the weighted average of at least 3 independent measurements averaged over 10 repetitions each.

#### 2.2.6. Fourier-Transform Infrared Spectroscopy by Attenuated Total Reflectance (FTIR-ATR)

The development of Fourier-transform infrared (FTIR) spectroscopy in both technological and digital aspects gives an increase in its applications. The attenuated total reflectance (ATR) technique adds at least one order in the sensitivity of the signal and eliminates the need for thick samples with sufficient content of the active substance for the transmittance measurements. Moreover, the large and intensive IR spectral bands of the water overlap with the bands of interest, and a distraction of the buffer spectrum is necessary. This diminishes drastically the photometric scale of the resulting effective spectrum. Therefore, we perform many scales to avoid the noise. All of these problems were met and overcome during the experiment reported here. Also, in all spectral sets, the spectrum of the pure POPC suspension with subtracted water spectrum is included for a comparison. FTIR spectra of the samples under investigation were collected with a Bruker, Vertex 70 spectrophotometer (Bruker, Rosenheim, Germany) in ATR geometry. A standard PIKE MIRacle ATR accessory equipped with a ZnSe reflection prism yielding 3 internal reflections and covered with a diamond plate was used. The range of interest 4000–600 cm^−1^ was limited at low frequencies by the transmission edge-cut of the ZnSe crystal. The spectra were obtained by averaging 100 interferograms with a resolution of 2 cm^−1^ at a temperature of 30 ± 0.3 °C maintained inside the instrument. The samples of water suspensions were filled into an o-ring (diameter 8 mm, thickness 1 mm) and put on the diamond plate, thus forming a basin.

## 3. Results and Discussion

### 3.1. FTIR-ATR Spectra Analysis 

A relatively selective “observation” of the hydrophobic chain dynamics and the perturbation on the polar hydrophilic heads was possible due to the spectral distance between the vibrational bands of the different parts of the lipid molecules [45,46,47]. The picture became clearer after removing the strong absorbing bands of the bulk water. The role of lipid hydration in the suspension is important for the bilayer state when interacting with the peptides, and this has been discussed widely [48,49,50]. The hydrogen-bond network of membrane-bound water shows a different bond orientation, which influences the place and the nature of the possible peptide–lipid interaction. Therefore, the spectra were carried out in situ with samples such as aqueous suspensions. 

Concerning the frequencies of the lipid vibrational modes, the authors are commonly in agreement [51,52]. Several distinct spectral regions are assigned to the specific vibrations in the different parts of POPC molecules. The spectrum at the highest frequencies of 2800–3600 cm^−1^ characterizes the C-H symmetric and asymmetric stretching vibrations in the hydrophobic chains, but the CH_3_ modes of the chains’ ends and the choline groups can contribute, too. Also, in this region, possible traces of the H-bond vibrations of the hydrated lipid have to be taken into account. In the spectral range of 1300–1600 cm^−1^ are the bands assigned to the numerous bending CH_2_ and CH_3_ modes in POPC. The lower frequencies 800–1300 cm^−1^ are characteristic of the vibrations in the headgroups at the membrane interface, as well as for rocking modes in the CH_2_ chains.

The measured spectra are presented and discussed in particular parts as they are mentioned above. These spectral domains avoid the ranges of the bending and libration modes of the bulk water molecules. Because of the small photometric values after the distraction of the water spectrum, just a noise was registered in the mentioned regions. An exception is the area, where the distraction reveals the specific spectral features of each sample, giving an idea about the mechanisms of molecular hydration.

#### 3.1.1. Spectral Region 3600–2800 cm^−1^

**Figure 2:**
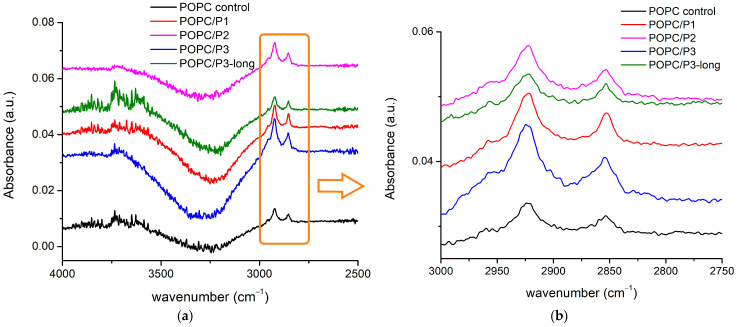
(**a**) FTIR-ATR spectra in the range of 4000–2500 cm^−1^ corresponding to C-H and OH vibrations; (**b**) absorbance in the spectral region of 3000–2750 cm^−1^ corresponding to CH_2_ and CH_3_ stretching vibrations (see text).

The FTIR-ATR spectra of all aqueous suspensions in this zone of the C-H, C-N, H-N and OH vibrational modes are presented in Figure 2. Looking at the higher frequencies in the spectral region of the OH vibrations, a very large band was registered (Figure 2a). This part of the spectrum gives information about the real molecular interactions in the aqueous suspension. 

The components arise as a result of the water’s interaction with the ingredients’ molecules. They can be assigned to the H-bond or to the OH-stretching vibrations in the vicinity of the different lipid molecular parts [49,50,51,52]. The intensive and structured band centered at 3247–3320 cm^−1^ most likely is a result of the intermolecular H-bonding near the lipid headgroups [50,51]. At the anionic phosphate group with a higher negative charge density than the oxygen atom of water, the negatively charged oxygen atoms directly form H-bonds with water which are stronger than that between water molecules. Replacing the alanine in the K by β-alanine yields some extra positively charged side chains in the peptide molecule. They can contribute to the formation of extra OH groups around the positive choline headgroups because of the hydrophobic character of the lipid–peptide interaction which leads to a break of the H-bond network in bulk water, thus giving rise to free OH vibrations in this spectral region. Indeed, the blue side of the broad band is most pronounced for the sample P3-long. A few weak components were registered around 3600–3720 cm^−1^, probably caused by the free OH vibration in the bulk water [50,52]. The most distinguished and relatively well-established bands are those of the CH_2_- and CH_3_-stretching vibrations in the hydrocarbon chains (Figure 2b). As the lipid membrane bilayer has a closely packed structure, an external perturbation can cause a detectable reflection, thus providing information about the motions of the hydrophobic tails. A conformational order–disorder state has been found to lead, respectively, to a downshift or upshift in the peak positions of the symmetric CH_2_ mode, while its half-width has been used as an indication of the degree of freedom of the tail motions [29,53,54]. The obtained peak positions and the bands’ half-widths of the corresponding symmetric and asymmetric CH_2_-stretching vibrations are summarized in Table 2. The addition of KLAKLAK-NH_2_ almost does not disturb the hydrocarbon backbone except for the slight narrowing of the band at 2853 cm^−1^. As the dipole moment of the CH_2_ symmetric vibration is in the C-C-chain plane [52], a negligible “hardening” can be assumed in this direction. All bands of CH_2_ vibrations for the POPC/P2 and POPC/P3 samples are significantly broadened by the rise of the CH_3_ symmetric and asymmetric vibrations at 2865 cm^−1^ and 2954 cm^−1^, respectively. The activation of the latter modes is probably caused mainly by the excitation of the CH_3_ choline groups. A possible explanation is given below. The end CH_3_ groups of the hydrocarbon chains also contribute as identified by the bands’ widening corresponding to tail agitating. Moreover, the deconvolution of both structured bands revealed that the CH_2_ sub-bands were broadened themselves (Table 2), which sustains the assumption of intensified tail motion. The doubled chain P3-long peptide samples do not exhibit considerable alteration in the hydrocarbon core movement but differ from the other spectra in the regions of the POPC polar group modes.

#### 3.1.2. Spectral Region 1750–1700 cm^−1^

This region consists of at least two bands originating from the two ester carbonyl groups in the lipid aggregate. They are usually revealed at high resolution [29,45,46,53,54]. The C=O-stretching modes are the most useful infrared bands for probing the membrane interface. The structural arrangement and environment of the lipid interfacial carbonyl groups are responsive to structural changes located at sites remote from the bilayer surface. Thus, the sensitive location of the C=O bonds between the polar headgroup and the nonpolar hydrocarbon chain gives a possibility for obtaining information about the changes in the chemical structure of lipid molecule parts.

**Figure 3:**
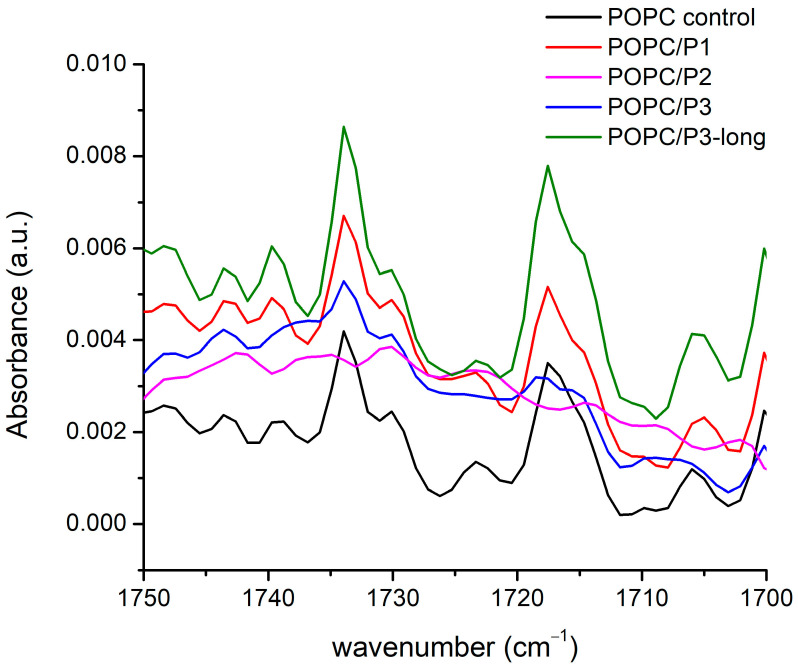
FTIR-ATR spectra in the range of 1750–1700 cm^−1^ corresponding to C=O-stretching vibrations in the glycerol esters.

In Figure 3, the FTIR-ATR spectra of all the samples in the vicinity of the two ester carbonyl groups’ C=O-stretching vibrations are given. More than two constituent bands were registered, but the main doublets were seen around 1733 cm^−1^ and 1717 cm^−1^, and their possible splitting depends on their hydration status [29]. The two bands are informative also because they are associated with the two lipid tails. It has been established that the bands at lower wave numbers are attributed to the hydrogen-bonded ester group, i.e., to the unsaturated lipid chain (sn2), while the band at a higher wave number indicates a lack of H-bonding and is associated with the saturated chain (sn1) [29,55]. Both modes have their polarization along the double bond [51], i.e., they have to be sensitive to external interactions along this bond. The spectra in Figure 3 certify the strongest P2 influence on POPC molecules in the polar regions surrounding the lipid carbonyls (most likely via protonation [52]); the two C=O bands completely vanish. A possible interpretation can be that the peptide’s cyclic rings are situated near the acyl chains, being sufficiently bulky to create steric disruption in the glycerol group, hence providing the acyl chains with more space to move but without inducing any indicative order or disorder in the core. Moreover, the increase in the bandwidth of the C-H-stretching vibrations registered and described in the previous subchapter indicates an increase in the fluidity of the bilayer [30]. A comparable model has been suggested in [53,56,57], investigating an intercalation of tricyclic antidepressants into phospholipid membranes by NMR. Some phenylalanines have been reported to be located in the lipid core, significantly disordering the hydrocarbon chains [58] but not disturbing the glycerol group, while other peptides point to the headgroups [59,60].

#### 3.1.3. Spectral Region 1600–1300 cm^−1^

**Figure 4:**
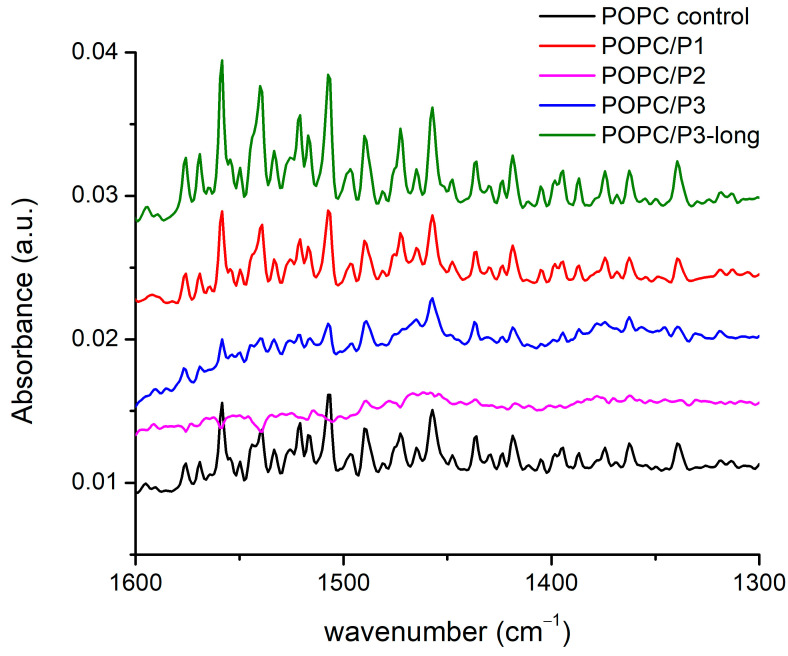
FTIR-ATR spectra in the range of 1670–1600 cm^−1^ corresponding to CH bending in the acyl chains’ and choline groups’ vibrations.

There are more than 20 registered modes in this region (Figure 4) arising from the CH_2_, CH_3_ and N-H-bending vibrations of different conformations including symmetric and asymmetric bond oscillations in and out of a specified plane, scissoring and waging, etc. [28,29,51,52,53,54]. Most of the bands overlap with one another, thus making difficult the exact assignment. Nevertheless, there are clear differences between the spectra: the lines are almost equivalent for the samples of pure POPC and POPC/P1, while for POPC/P3-long the modes become visibly more intensive and slightly narrower. The bands in the spectrum of the sample POPC/P3 diminish in intensity and are enlarged. The spectrum of POPC/P2 is almost flat in this region. 

As the modes originate mainly from the bonds in the lipid chain, they characterize their state in the various environments, although at these frequencies the umbrella-like vibrations of the CH_3_ of the choline group are active too. The polarization of the bending vibrations in the chain is perpendicular to the backbone; the polarization of the choline-end vibrations depends on the symmetry and is ⊥ or || to the local C_3_ axis for the A_1_ or E type, respectively [51]. Consequently, the properties of the CH_2_ bending are determined by the lateral packing interactions between the chains. Deviations of the hydrocarbon chain conformation from all-trans lead to marked alterations in the spectrum, such as loss of intensity, band broadening and finally the disappearance of the modes and vice versa—any parallel coupling with this kind of vibration increases the resulting oscillating dipole moment, i.e., the intensity of the absorption bands. The results in Figure 4 identify three samples to fit these trends in the spectra. Moreover, again as pointed out above, the P2 analog alters the lipid bilayer relatively in the highest degree. Probably, the bulky cyclic rings disturb the chains up to the loose of the symmetry of this type of vibration, which are usually weak. It is noteworthy that the band at 1472 cm^−1^ completely disappears in the spectra of the samples POPC/P2 and POPC/P3. This mode is assigned to the asymmetric N-CH_3_ vibrations in the choline group [51] but overlaps also with the N-H-bend and C-N-stretching vibrations of the peptide’s Amide II [52].

#### 3.1.4. Spectral Region 1300–800 cm^−1^

**Figure 5:**
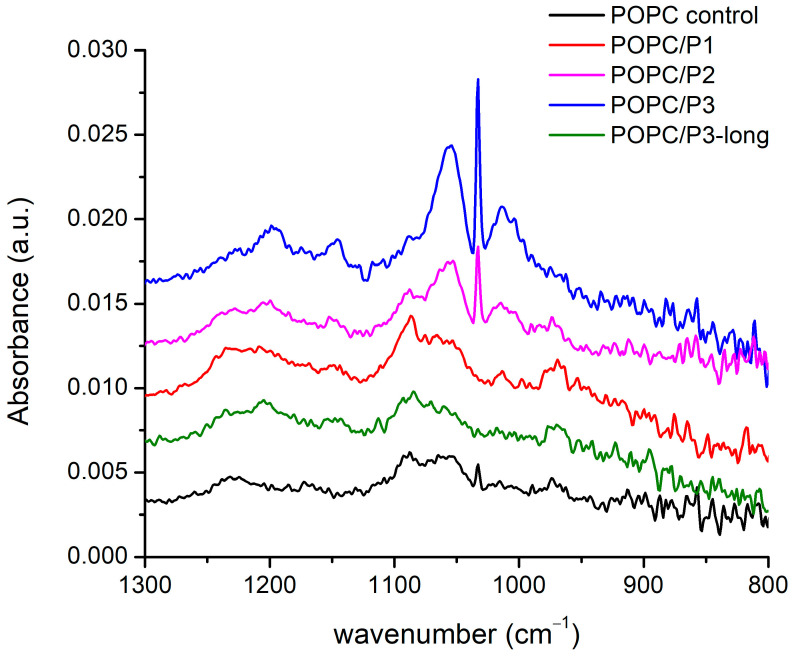
FTIR-ATR spectra in the range of 1300–800 cm^−1^ corresponding to phosphate and choline groups’ vibrations.

The spectral alterations as a result of POPC–peptide interactions are displayed in Figure 5 in the range of the active vibrations in the lipid headgroup. The modes include the usual low-frequency modes of oscillation in phosphate and choline groups: twisting, rocking, etc. The phosphate group is generally monitored via the O-P-O antisymmetric stretching mode, which is seen as a very broad band of about 1250–1150 cm^−1^. The phosphate group provides hydrogen-bonding acceptors, leading to a dependence of the spectral position on the hydration state of the headgroup: upon hydration, the asymmetric O-P-O stretching frequency decreases [29,51], whereas the broadening of the band is an indicator of polar headgroup mobility in the presence of the peptide [47]. In the complicated structure of this large band, a frequency decrease in the main peak of the asymmetric double-bond stretching O-P-O, adopting a maximum from 1229 cm^−1^ to 1197 cm^−1^, is seen through the samples. The lowest frequency is obtained for the most active peptide with β-alanine in the structure (P3).

The band at 1086 cm^−1^ arising from the symmetric O-P-O double-bond stretching vibrations is narrower and does not alter in frequency. The dipole moment of oscillations is along the bisector of the angle of deviation, and a possible coupling with the positively charged side chains of P1 leads to an increase in the intensity of this band. For the P3 analog, the mode vanishes while it remains active for the asymmetric vibrations with perpendicular polarization towards which the protonation process probably occurs. The few near bands at 1056 cm^−1^, 1033 cm^−1^, 1014 cm^−1^ and 971 cm^−1^ concern the C-O, CNC and P-O single-bond stretching vibrations in the headgroup. The best polar coupling with the first three modes in the direction perpendicular to their bonds is observed for P2 and P3. The last band with an opposite dipole moment vanishes in the spectrum of POPC/P3. It has to be underlined that the mode of the asymmetric N-CH_3_-bending vibration in the choline group discussed in the previous subsection becomes inactive in the spectra of both mentioned samples. 

In the presented region, the strongest influence of the P3 peptide on the POPC headgroup was registered. Like in Section 3.1.1, the same possible model of molecular relations can be adopted. The substitution by β-alanine of the alanine in KLAKLAK-NH_2_ supplies the peptide with some extra positively charged side chains which sustains the idea that longer molecules assume a different orientation in the membrane, presumably lateral, thus decreasing the interaction activity. Indeed, the spectra of P3 and P3-long are almost identical to that of the pure POPC in all the considered regions.

#### 3.1.5. Spectral Region 1600–1670 cm^−1^

Lipid–peptide interactions result also in alterations in the peptide spectra measured. When in contact with membranes, most of the peptides adopt an amphipathic arrangement that contains α-helical, β-sheet, or both structures. Examining the interactions between the peptides and host membranes, infrared spectra show possible conformational changes in the different samples. The clarifying of the problem becomes difficult because of the very low peptide concentrations, which results in very weak bands. Moreover, the amide modes often coincide with those of the complex lipid aggregate in the region of the bending vibrations. Nevertheless, a few bands were found to be informative about the form of the mutual interactions. The amide I band indicates a strong hydrogen bonding in the α-helical peptide, which is sensitive to membrane fluidity, i.e., to the lipid chain mobility discussed above [52]. Several distinct structures of the peptide–lipid binding have been observed, suggesting that the peptides undergo functionally relevant changes in their conformation and membrane alignment [53,61,62,63,64]. More generally, surface S-states and transmembrane T-states have been reported, respectively, with a parallel and oblique direction of the helix axis toward the normal to the bilayer surface. It is useful to have in mind the difference in the abilities of the interaction of the complete helix and of the peptide local side chains. The capacity of the latter to hydrogenate the lipid headgroups is discussed largely in the previous subchapters.

**Figure 6:**
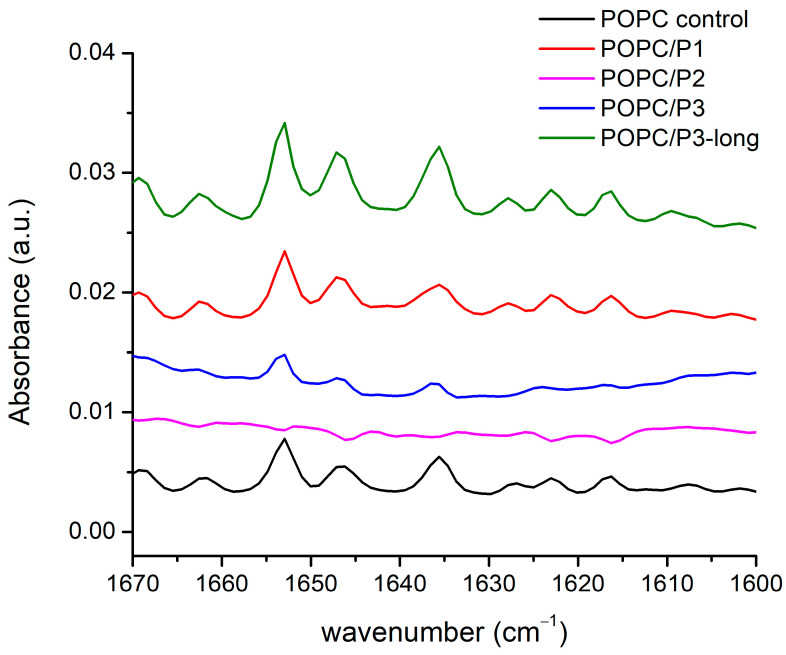
FTIR-ATR spectra in the range of 1670–1600 cm^−1^ corresponding to the peptides’ Amide I, α-helix and β-sheets modes.

FTIR-ATR spectra of the aqueous suspensions according to the legend in the zone of the peptides’ Amide I, α-helix and β-sheets’ modes are presented in Figure 6: the doublet 1660–1640 cm^−1^ is assigned to Amide I of the α-helix, and the band at 1635 cm^−1^ is assigned to the Amide I of the β-sheets [52]. As it is seen, all three bands vanish completely for the sample POPC/P2. The analysis of the spectra shown above suggests a “soft” penetration of the phenyl rings of 1,8-naphthalimide in the lipid hydrophobic area thus presuming the oblique orientation of the helix correlating with the vibrational polarity in the choline group leading to the strong narrow peak at 1033 cm^−1^ (see Figure 5). This peak is maximal for the POPC/P3 sample, in which the maximal mobility in the lipid chains is registered, and it is not caused by peptides penetrating the core through the lipid interfacial carbonyl groups but probably by the strong electrostatic interaction in the headgroup vicinity. In Figure 6, this sample exhibits diminishing amide vibrations like in Figure 3 and Figure 4. In the POPC/P3-long samples, the spectral regions in Figure 3, Figure 4 and Figure 6 testify to a probable lateral association to lipid molecules without altering significantly the main lipid bilayer organization. 

### 3.2. Bending Elasticity of POPC Membranes in the Presence of KLAKLAK-NH_2_ and Its Analogs

For each peptide studied, we determined the total peptide-to-lipid ratio (P/L) at which fluctuating GUVs without membrane defects were formed to satisfy all TSFA criteria discussed in detail previously [41,65]. The total lipid concentration in the experiments with GUVs ranged between $C_{L}^{tot}$ = 33 µM and 37 µM. The highest P/L that allows for producing flaccid quasispherical GUVs was achieved for KLAKLAK-NH_2_ (P1) and for NphtG-KLβAKLβAK-NH_2_ (P2)-containing samples. At very high KLAKLAK-NH_2_ (P1) concentrations (~30 µmol/L) and a total peptide-to-lipid molar ratio P/L~0.90 mol/mol, we observed only aggregates after electroformation. The lipid stacking originates from the combination of the apparent negative charge of the phosphatidylcholine bilayers due to the surrounding water dipole arrangement [66] in water and the presence of two positively charged lysine residues in P1, engendering electrostatic interactions between the peptide molecules and the POPC membranes.

**Figure 7:**
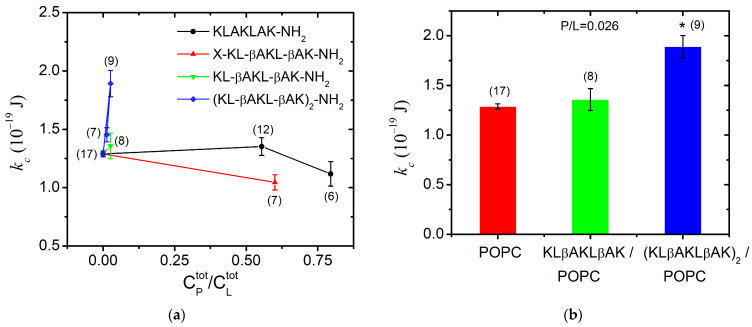
Bending elastic modulus (weighed mean ± SD) of POPC membranes in the presence of (**a**) KLAKLAK-NH_2_ and its analogues at different total peptide-to-lipid molar ratios in the sample; (**b**) β-alanine containing peptides KLβAKLβAK-NH_2_ and (KLβAKLβAK)_2_-NH_2_ at 0.026 total peptide-to-lipid molar ratio (the number of GUVs given in brackets above each data point/column; * *p* < 0.005, $\chi^{2}$-test of independence from the control).

The bending rigidity of the POPC bilayers was obtained at two P1 concentrations near the lowest concentration at which the peptide has exhibited in vitro antiproliferative and cytotoxic activity [18]. At P1 content P/L~0.80, we measured membrane softening with a nearly 15% reduction in the bending modulus in comparison to POPC membranes as shown in Figure 7a. This finding suggests the surface orientation of the peptide at the bilayer as reported in [67] for alamethicin, which is an antimicrobial peptide much more hydrophobic than KLAKLAK-NH_2_. Hence, very low total concentrations of alamethicin such as ${P/L<10}^{-3}$ mol/mol produce significant membrane softening [67]. In the case of P1, we report only a slight reduction in the bending rigidity of POPC bilayers at nearly-three-orders-of-magnitude-higher P1 content in the sample, which we attribute to the significantly lower partition coefficient of P1 between the bilayer and the surrounding water [68]. The primary structure of P1 contains a doubled sequence of three amino acids, including the charged lysine and the non-polar leucine and alanine. This amphipathicity underlies the peptide partitioning between the lipid bilayer and the aqueous environment [69]. As obtained from the FTIR-ATR spectra analysis of POPC/P1 samples at P/L~0.2, the non-modified peptide only negligibly disturbs the hydrocarbon backbone, which corresponds to our findings from TSFA about the unaltered bending elasticity of POPC bilayers in the same concentration range of P1 (Figure 7a).

The KLAKLAK-NH_2_ analog P2 containing 1,8-naphtalimide and β-alanines in place of alanines also induced membrane softening. Fluctuating quasispherical GUVs were readily obtained from POPC/P3 mixtures at a relatively high peptide concentration, P/L= 0.6 in the sample. The bending modulus was measured to decrease by nearly 20% as shown in Figure 7a. Statistical models developed recently [70,71] have considered the interactions of membrane-incorporated peptides, mediated by membrane elastic deformations. The bending elasticity changes are related to the lateral redistribution of the peptide molecules, incorporated in the lipid matrix as well as to their ability to induce spontaneous curvature in the bilayer [72,73,74,75]. The most considerable alteration of the membrane-bending rigidity reported for P2 is in agreement with the FTIR-ATR spectroscopy findings in the spectral region 1750–1700 cm^−1^ about the P2 influence on POPC molecules in the polar region surrounding the lipid carbonyls. The latter is attributed to the peptide’s cyclic rings, which are sufficiently bulky to induce steric disruption in the glycerol group. Evidence indicating increased bilayer fluidity is provided by the spectral characteristics in the region of 1600–1300 cm^−1^ as discussed in the previous subsection. The reported membrane softening is further corroborated by the increased fluidity of the bilayer detected from the bandwidth of the C-H-stretching vibrations’ signature in the spectra of POPC/P2 samples. 

For KLβAKLβAK-NH_2_ (P3), the GUV formation was hindered at much lower total peptide-to-lipid ratios P/L≥ 0.33, producing stuck lipid–peptide structures and foam-like aggregates. As reported above from FTIR-ATR spectroscopy data, the hydrophobic lipid tails of POPC/P3 samples (P/L~0.2) exhibit high mobility with no evidence of peptides penetration into the core through the lipid interfacial carbonyl groups but suggesting strong electrostatic interaction in the headgroup vicinity (spectral region 1600–1670 cm^−1^). After decreasing the peptide content in the sample, small non-fluctuating GUVs started forming at P/L = 0.11. Further reducing P/L down to 0.026 led to the formation of flaccid quasispherical GUVs with diameters above 10 µm, allowing the thermal shape fluctuation analysis to be successfully applied for the determination of the bending modulus, which remains unaltered compared to its value for pure POPC membranes (Figure 7a). 

The presence of the doubled P3-long sequence rigidified the lipid bilayer even at very low total concentrations of P/L~0.03. A steep growth of the bending constant is reported with the increase in peptide concentration in the sample as shown in Figure 7a. This finding is in accordance with the results from the FTIR-ATR spectra analysis (spectral region 1300–800 cm^−1^), revealing the strong effect of the P3 peptide on lipid headgroups. The substitution by β-alanine of the alanine in KLAKLAK-NH_2_ provides some extra positively charged side chains, suggesting a peripheral orientation on the membrane. The effects of P3 and P3-long on the membrane’s mechanical properties are juxtaposed in Figure 7b for P/L~0.03. The doubled P3-long analog rigidifies more strongly the bilayer in agreement with the findings from FTIR-ATR spectroscopy data, suggesting a probable lateral P3-long association to lipid molecules without significant alteration of the main lipid bilayer organization.

### 3.3. Impedance Characteristics of Lipid Bilayers in the Presence of KLAKLAK-NH_2_ and Analogues

The electrical parameters of suspended planar POPC bilayers, including their capacitance and resistance, were measured using FFT-EIS to probe for non-specific interactions of KLAKLAK peptides with the lipid matrix of the membrane.

**Figure 8:**
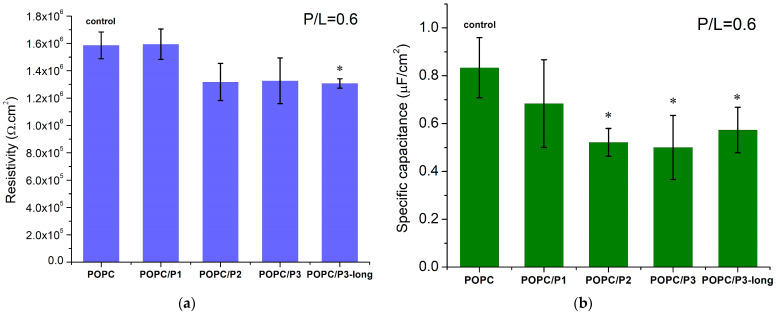
(**a**) Resistivity and (**b**) specific capacitance of POPC bilayers in the presence of KLAKLAK-NH_2_ and its analogues at 0.6 total peptide-to-lipid molar ratios in the samples; values presented as weighted mean ± SD; at least three independent measurements (averaged over 10 repetitions each; * *p* < 0.005, $\chi^{2}$-test).

For a given peptide analog, at least three BLMs were prepared and impedance measurements were performed, with each of them averaged in over 10 repetitions. The weighted mean values with the corresponding standard deviations are presented in Figure 8. The POPC membrane resistivity obtained from the equivalent circuit fitting of the experimental data is coherent with the values for phosphatidylcholine bilayers, measured so far, $R_{m}$~10^6^ Ω cm^2^ [34,76,77]. KLAKLAK-NH_2_ does not exert a measurable effect on the membrane resistivity, whereas all β-alanine-containing sequences induce a slight reduction in this parameter (Figure 8a). The substitution of alanines by β-alanines imparts additional positively charged side chains in the peptide molecule. Therefore, the formation of extra OH groups is expected around the positive choline headgroups as discussed above. A lower specific capacitance of KLAKLAK-treated POPC bilayers was measured for all peptide analogs at 0.6 peptide-to-lipid molar ratios in the samples (Figure 8b). While the obtained decrease for P3- and P3-long-treated membranes might be related to bilayer thickening, the reduced capacitance of P1- and P2-containing samples is likely due to lower dielectric permittivity. As reported from FTIR-ATR spectra, the bulky naphtalimid cyclic ring in the P2 molecule alters the aliphatic chain order in the hydrophobic core of the bilayer, which likely changes the dielectric permittivity of the lipid membrane.

## 4. Conclusions

The findings reported above elucidate the effect of the KLAKLAK-NH_2_ peptide family on important physicochemical parameters of lipid membrane models. Among others, the membrane-mediated processes in the biological cell are strongly related to the membrane-bending rigidity, one of the properties studied here. The specific capacitance and resistivity of lipid bilayers were explored in the presence of the antimicrobial peptide mimetics in view of unravelling their impact on membrane impedance characteristics. The latter are relevant to deciphering the effect on the electrical properties of lipid bilayers in order to shed light on the governing mechanisms involved in the transmission of electrical impulses or electroporation-based applications. The Fourier-transform infrared spectra with attenuated total reflectance revealed the interaction of KLAKLAK-NH_2_ and KLβAKLβAK-NH_2_ analogs with the polar head region of lipid bilayers. A similar effect on the mechanical properties of POPC membranes is obtained upon treatment with β-alanine-substituted short and long KLAKLAK-derivatives. The FTIR-ATR spectra of POPC bilayers in the presence of the long amino acid sequence with the β-alanine substitution of alanine lead to the conclusion of a probable lateral peptide association to the membrane. This finding is consistent with the results from the thermal shape fluctuation analysis, which reveal the concentration-dependent higher bending rigidity of (KLβAKLβAK)_2_-NH_2_-treated membranes. The membrane thickening amounts to lowering the specific electrical capacitance of KLβAKLβAK-NH_2_ and (KLβAKLβAK)_2_-NH_2_ containing samples. The bonding of 1,8-naphthalimide to the KLβAKLβAK-sequence induces the relatively most-pronounced structural alteration, which corroborates with the reported membrane softening. 

The introduction of the β-amino analog of Ala into the primary structure of KLAKLAK-NH_2_ peptides producing more rigid and conformationally confined amphiphilic structures (P2, P3 and P3-long peptides) results here in the most important alterations of the physicochemical properties of the lipid membrane. These alterations are related to the antiproliferative and antimicrobial activity of the studied peptides reported so far [18,19]. The short P3-analog has been shown to exhibit a high antiproliferative effect against mammary gland type A adenocarcinoma cells (MCF-7) [18], while the doubled P3-long peptide combines low cytotoxicity and high antibacterial activity [19]. The slight nonselective increase in the biological activity of the P2 analog upon the bonding of the additional group 1,8-naphtalimideG [18] is coherent with the above findings about the structural and mechanical alterations of the lipid bilayer. 

The obtained results present comprehensive information on the molecular interactions of the studied peptide mimetics on lipid membrane models. The reported influence on the structural, mechanical and impedance features of lipid membranes provides a useful groundwork for the development of liposome-based formulations for drug delivery and biomedical applications.

## Figures and Tables

**Table 1:** Description of the peptide sequences.

Peptide	Structure	Molecular Mass (g/mol)
P1	KLAKLAK-NH_2_	769.52
P2	* NphtG-KLβAKLβAK-NH_2_	1006.56
P3	KLβAKLβAK-NH_2_	769.52
P3-long	KLβAKLβAKKLβAKLβAK-NH_2_	1522.05

* Npht denotes 1,8-naphthalimide.

**Table 2:** Spectral regions of the stretching C-H vibrations.

	Sample	POPC	POPC/P1	POPC/P2	POPC/P3	POPC/P3-Long
Parameter						
Peak wavenumber, cm^−1^		2922 asym CH_2_	2922 asym CH_2_	2922 + 2954 asym CH_2_ + CH_3_	2922 + 2954 asym CH_2_ + CH_3_	2922 asym CH_2_
Half-width, cm^−1^		19	20	31	30	24
Peak wavenumber, cm^−1^		2853 sym CH_2_	2853 sym CH_2_	2853 + 2865 sym CH_2_ + CH_3_	2853 + 2865 sym CH_2_ + CH_3_	2853 sym CH_2_
Half-width, cm^−1^		16	13	17 (24)	19 (30)	14

## Data Availability

Small amounts of the studied compounds are available from the corresponding author. All raw experimental data and protocols are also available from the corresponding authors.
